# Inhibition of the Nogo-pathway in experimental spinal cord injury: a meta-analysis of 76 experimental treatments

**DOI:** 10.1038/s41598-023-49260-5

**Published:** 2023-12-21

**Authors:** Julian Hirt, Alireza Khanteymoori, Marc Hohenhaus, Marcel A. Kopp, David W. Howells, Jan M. Schwab, Ralf Watzlawick

**Affiliations:** 1 Department of Neurology and Experimental Neurology, Charité Campus Mitte, Clinical and Experimental Spinal Cord Injury Research Laboratory (Neuroparaplegiology), Charité – Universitätsmedizin Berlin, Berlin, Germany; 2 Department of Neurosurgery, Medical Center - University of Freiburg, Faculty of Medicine, University of Freiburg, Breisacher Straße 64, 79106 Freiburg, Germany; 3 Faculty of Medicine, Dentistry and Health Sciences, The University of Melbourne, Melbourne, VIC Australia; 4 Department of Neurology, Spinal Cord Injury Division (Paraplegiology), The Neurological Institute, The Ohio State University, Wexner Medical Center, Columbus, OH USA; 5 Belford Center for Spinal Cord Injury, Departments of Neuroscience and Physical Medicine and Rehabilitation, The Neurological Institute, The Ohio State University, Wexner Medical Center, Columbus, OH USA

**Keywords:** Neuroscience, Medical research, Neurological disorders, Nervous system

## Abstract

Recovery after spinal cord injury (SCI) may be propagated by plasticity-enhancing treatments. The myelin-associated nerve outgrowth inhibitor Nogo-A (Reticulon 4, RTN4) pathway has been shown to restrict neuroaxonal plasticity in experimental SCI models. Early randomized controlled trials are underway to investigate the effect of Nogo-A/Nogo-Receptor (NgR1) pathway blockers. This systematic review and meta-analysis of therapeutic approaches blocking the Nogo-A pathway interrogated the efficacy of functional locomotor recovery after experimental SCI according to a pre-registered study protocol. A total of 51 manuscripts reporting 76 experiments in 1572 animals were identified for meta-analysis. Overall, a neurobehavioral improvement by 18.9% (95% CI 14.5–23.2) was observed. Subgroup analysis (40 experiments, N = 890) revealed SCI-modelling factors associated with outcome variability. Lack of reported randomization and smaller group sizes were associated with larger effect sizes. Delayed treatment start was associated with lower effect sizes. Trim and Fill assessment as well as Egger regression suggested the presence of publication bias. Factoring in theoretically missing studies resulted in a reduced effect size [8.8% (95% CI 2.6–14.9)]. The available data indicates that inhibition of the Nogo-A/NgR1pathway alters functional recovery after SCI in animal studies although substantial differences appear for the applied injury mechanisms and other study details. Mirroring other SCI interventions assessed earlier we identify similar factors associated with outcome heterogeneity.

## Introduction

**Figure 1:**
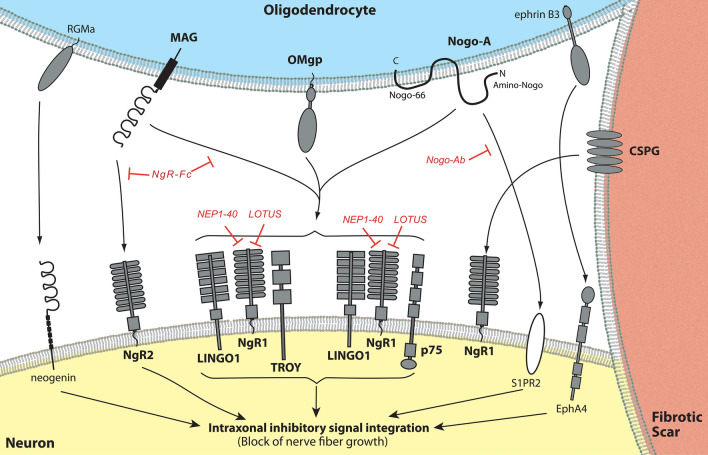
Nogo-A signaling restricting axonal outgrowth. After SCI nerve growth inhibitory molecules from oligodendrocytes interact with various receptors on injured neurons within the Nogo-pathway^29^. Nogo-A, oligodendrocyte-myelin glycoprotein (OMgp) and myelin-associated glycoprotein (MAG) exert signaling mainly through the Nogo Receptor 1 (NgR1) which can form a receptor complex with leucine rich repeat and Immunoglobin-like domain-containing protein 1(LINGO1), and p75^NTR^ or TROY. Additional signaling from repulsive guidance molecule–A (RGMa), ephrin B3 and the N-terminal domain of Nogo-A (Amino-NOGO) interact with other neuronal receptors (NgR2, neogenin, EphA4, S1PR2^30^). Downstream signaling results in RhoA/ROCK-activation (Ras homolog family member A / Rho-kinase) and the block of nerve fiber growth^31^. Possible therapeutic interventions targeting the Nogo-signaling are shown in red. (Modified form^1,5^).

Spinal cord injury (SCI) leads to destruction of neurons, astrocytes and oligodendrocytes disrupting ascending and descending axonal tracts. In contrast to the peripheral nervous system, central nervous system (CNS) axons regenerate far less after injury. Axonal outgrowth in the adult CNS is hindered by both intrinsic and extrinsic factors. Extrinsic nerve fiber growth impediments include myelin debris, reactive astrocytes and scarring fibroblasts^1–6^. In the 1980s the first molecules underlying myelin inhibition blocking neurite outgrowth were identified^2,7,8^ (Fig. 1). The first myelin associated nerve growth inhibitor (NGI) has been characterized and referred to as Nogo-A^9^, as a member of the reticulon family^10,11^. Other NGI were discovered subsequently, namely the myelin associated glycoprotein (MAG)^12^ and oligodendrocyte-myelin glycoprotein (OMgp)^13,14^. These three NGI were shown to directly bind to two receptors on CNS axons: the Nogo-66 receptor NgR1 and the paired immunoglobulin-like receptor B (PirB)^8,15^. At the moment much less is known about PirB but NgR1 has been extensively studied^8^. NgR1 can form a receptor complex with several possible coreceptors such as LINGO-1^16^, p75^17^ and TROY^18^. Of note NgR1 is a receptor for the plasticity restricting chondroitin sulphate proteoglycans (CSPGs)^19^ which are localized in the reactive astrocytes. MAG also binds to the NgR2 receptor^20^. Other outgrowth blocking molecules localized in the myelin, among others, include EphrinB3 and the Repulsive Guidance Molecule a (RGMa)^21,22^, which bind to EphA4 or neogenin respectively^23–25^. Intraaxonal integration of inhibitory molecular cues result in abrogation of neuroaxonal sprouting responses^5,26^. Additional reports indicate that blocking the Nogo-A pathways can also exert vasculoprotective and vasculogenic functions^27,28^.

Experimental SCI models reported propagated axonal outgrowth and motor recovery through inhibition of the Nogo-A/receptor pathway^1,32^. Results from large animal models applying Nogo-A antibody treatment of SCI verified plasticity-enhancing effects^33^ and led to the development of the human antibody ATI355. A phase I clinical trial of ATI355 demonstrated safety after intrathecal application in human SCI patients^34^ and a phase II clinical trial is currently underway. A complementary interventional approach applies a human fusion protein targeting the Nogo receptor (NgR) to mitigate myelin-associated axonal outgrowth signalling operated through the ligands Nogo-A, MAG, and OMgp^35^. The NgR1 receptor blocker Axer 204 has been tested in early first-in-man studies recently^36^.

Recent reports indicate that the predictive value in experimental SCI studies can be undermined by underestimated variability and overstated effectiveness^37^. As the field of SCI progresses towards interventional testing we conducted a systematic and meta-analytic (including Funnel plotting, Egger regression & trim and fill method) assessment of effect sizes associated with various strategies interfering with the Nogo-A pathway to inform the translational process.

## Results

### Study selection

**Figure 2:**
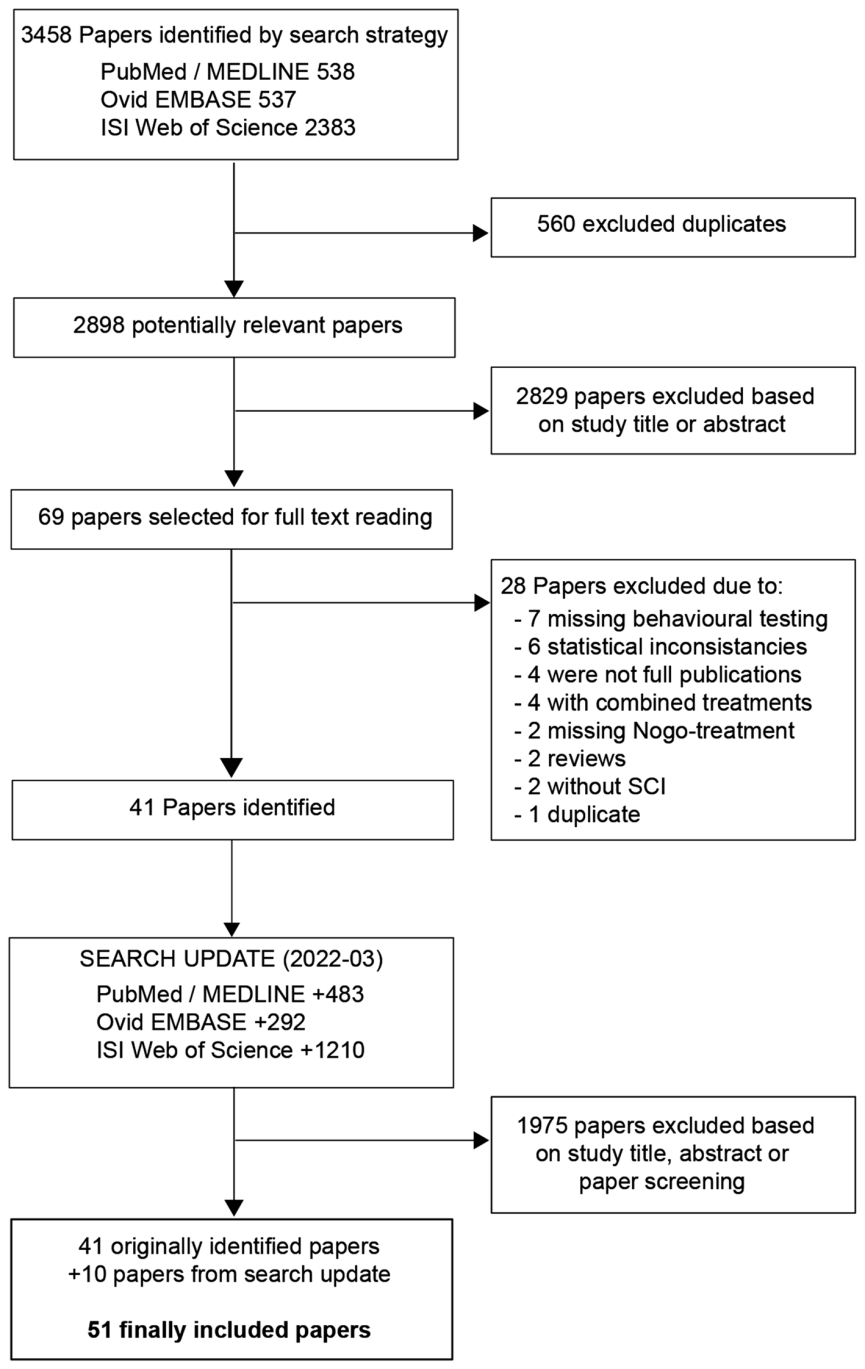
Study selection. The performed literature research interrogating several data bases (PubMed, Ovid, ISI Web of Science) identified 5443 studies where 51 studies were selected for the final data analysis. The analysis focused on studies with reported motor outcomes.

The initial search identified 3458 studies, of which 560 were immediately excluded due to them being replicates in the multiple data bases searched (Fig. 2). A further 2829 studies were excluded because of lack of relevance based on title and abstract after screening by two reviewers, leaving 69 for closer inspection. Of these 69 publications, 41 met the inclusion criteria and were included into this meta-analysis. Seven studies were excluded as they reported no behavioural testing, six because they had statistical inconsistencies, four were not full publications with missing study details, four only reported combined treatment, two did not inhibit the Nogo-A pathway, two were reviews, two did not injure the spinal cord and one was a duplicate publication (same data published in two different publications, one was included). The search update in 2022 added 1985 studies of which 10 additional studies were included. For the final analysis 51 studies with 76 experiments and 1572 animals were included (Supplemental Table 1).

### Characteristics of studies

Overall, 76 experiments were conducted in different mammalian species (46 in rats, 26 in mice and 4 studying non-human primates). Four different types of injury were observed: 52 experiments performed spinal cord hemisection, 15 experiments reported contusion injuries, 8 experiments performed complete transections and one experiment used compression injury. The most commonly used intervention was the application of Nogo-A-Antibodies (28 experiments) followed by NgR-blockers (31 experiments) and Nogo or NgR-knockout animals (NgR/Nogo-knockout, 17 experiments). Concerning neurological outcome, the BBB scale^38^ was used in 51 experiments. 12 experiments utilized the BMS scale^39^ and 13 experiments used other neurological scales. All studies injured the spinal cord in either cervical or thoracic segments with 7 experiments applying damage to the cervical and upper thoracic spinal cord (C3–T2) and 69 experiments applying damage to the thoracic spinal cord (T6–T10).

### Overall neurobehavioral outcome

**Figure 3:**
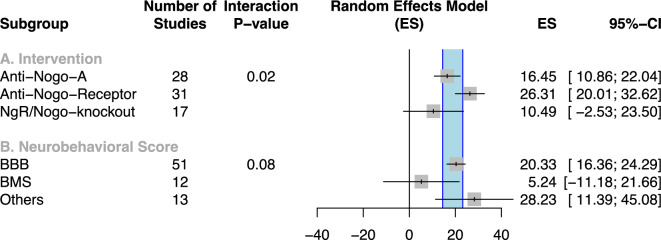
Stratified meta-analysis for the overall dataset (all outcome parameters). The reported overall effect size was 18.9% (95% CI 14.5–23.2) indicated by the blue shaded area within the forest plot. Several aspects of the study design accounted for ‘between-study’-heterogeneity such as drug target/intervention, chosen SCI modality and corresponding neurobehavioral scale. (**A**) Interventions blocking the Nogo-receptor indicated the highest effect sizes within the overall dataset. (**B**) The most commonly used score for neurobehavioral assessment was the BBB-score in 67% of all included experiments. “Others” subsumes primate data revealing the largest effect sizes likely attributable to more subtle transection lesions with larger spared plasticity reservoirs. *(ES: effect size, CI: confidence interval, BBB: Basso, Beattie, and Bresnahan score*^38^*, BMS: Basso Mouse Scale*^39^*).*

Inhibition of the Nogo-pathway led to improvement of neurobehavioral outcome by 18.9% (95% CI 14.5–23.2) within the randomised effects model. Stratified meta-regression of the entire data set revealed that “Drug group” had a significant impact on study heterogeneity with the effects of Nogo-A and NgR-receptor having the greatest efficacy (*p *= 0.02, Fig. 3A). Stratification for neurological recovery suggested that response to treatment in mice (assessed by BMS^39^) is lower [effect size] as compared with rats (assessed by BBB scale^38^) (*p *= 0.08, Fig. 3B). Primates are clustered in the category “others”. It is noteworthy that while recovery was the highest the variance detected in these studies was also highest. To explore reasons contributing to outcome heterogeneity, a subgroup analysis was performed comprising the largest and most homogeneous data (smallest CI, Fig. 3B) cohort of experimental SCI in rats (BBB Score). Since the BBB-score has been applied for neurobehavioral testing in some mice experiments (11 experiments), these experiments were also excluded from the subgroup analysis.

### Subgroup analysis

**Figure 4:**
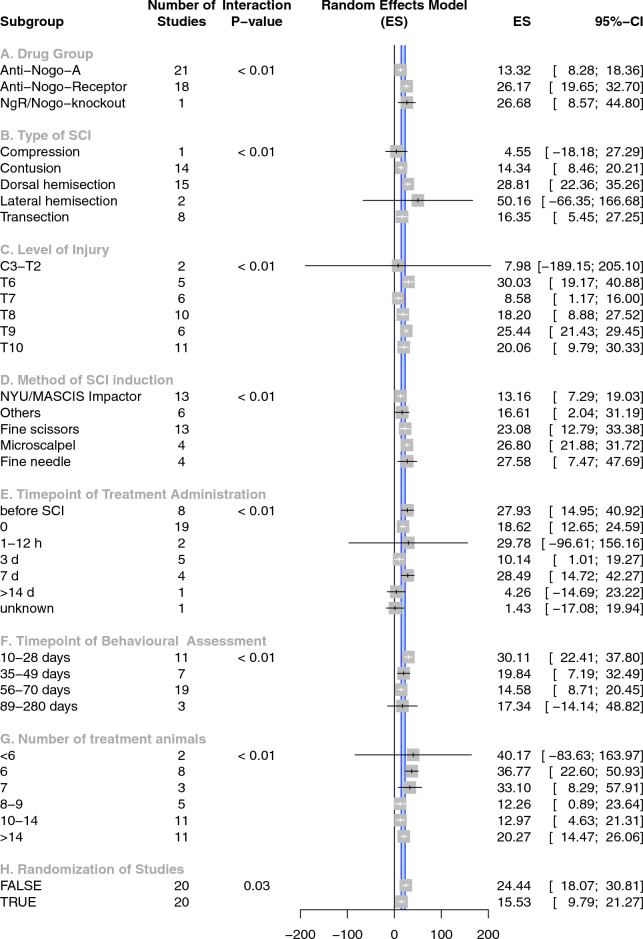
Effect of study characteristics on locomotor recovery. Differential effects of SCI modelling on neurological recovery were assessed by including only studies restricted to rats applying the BBB-score (40 experiments containing 890 rats) to increase the level of homogeneity (stratified meta-analysis). The blue shaded area within the forest plot represents the 95% CI limits of the global estimate of efficacy: 18.7% (95% CI 14.4–23.0). The horizontal error bars represent the 95% CIs for the individual estimates. Each stratification accounts for a significant proportion of between-study heterogeneity *(ES: effect size, CI: confidence interval).*

**Figure 5:**
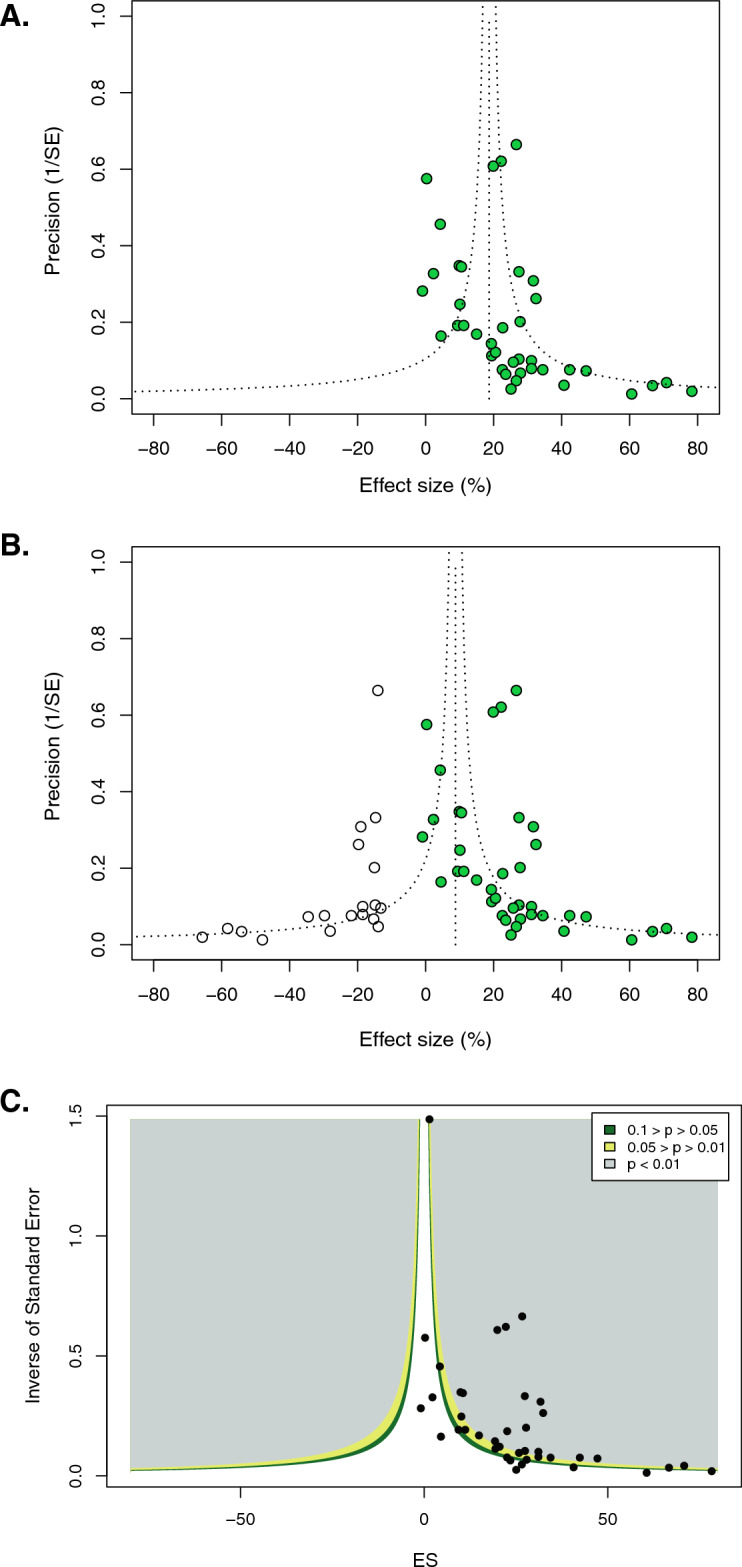
Empirical evidence of missing data (data asymmetry). (**A**) Funnel plotting of the rat (BBB-only) cohort including 40 included experiments (n = 890) reported an overall neurobehavioral efficacy of 18.7% (95% CI 14.4–23.0). (**B**) Trim and fill analysis added 19 experiments (white circles) indicating missing data. Factoring in missing data points resulted in a reduced effect size of 8.8% (95% CI 2.6–14.9). (**C**) Contour-enhanced funnel plot adds three shaded areas of statistical significance to the funnel plot. Most of the experiments lie within the grey area (highly significant results *p *< 0.01) or within the light green area (*p *< 0.05). Visual inspection for plot symmetry would impute potential missing studies close to the threshold of significance on the left side of the funnel plot or within the grey area of statistical significance^40^.

To exclude the skewing effects of variable outcome tests and different species, we focused on data applying the most prevalent SCI recovery assessment, the BBB score. The BBB has been developed to study for locomotor recovery after SCI in rats. Experiments applying outcomes other than the BBB-score or using animals other than rats were excluded. This provided a large homogenous subgroup containing data from 40 experiments containing 890 rats (802 Sprague–Dawley, 46 Lewis, 42 Longs-Evans) (Fig. 4). The random effect model indicated an overall effect size of 18.7% (95% CI 14.4–23.0) for the included experiments (Fig. 5). Stratified meta-regression indicated substantial differences between ten subgroups (Fig. 4).

A. Intervention: Overall when assessing interventions in SCI rat models using BBB outcome assessments, strategies applying Nogo-A antibodies demonstrated a 13.3% improvement. Blockade of the NgR resulted in an effect size of 26.2%. Genetic knock-out approaches either deleting NgR or Nogo-A regions revealed an effect size of 26.7% (*p *< 0.01, Fig. 4A).
B. SCI Type: Static compression models resulted in the smallest effect sizes compared to transection and contusion models. Hemisection cord injury permitted the greatest intervention efficacy, especially for lateral hemisections (*p *< 0.01, Fig. 4B). However, the stratification for complete and incomplete SCI showed an improved effect size of 5.3% in favour of incomplete studies but this stratification did not account for statistical significance (data not shown).
C. Lesion level: More than half of the experiments induced thoracic SCI lesions at the level T8–T10. These showed comparable and significantly beneficial effect sizes. Cervical and high thoracic injuries displayed the highest variability (*p *< 0.01, Fig. 4C).
D. Sub-specification of SCI injury modality: Method of injury induction: SCI induced by impactors inflicted a deficit which achieved the smallest motor recovery. Use of a fine needle to cut the spinal cord allowed the largest amount of recovery consistent with a smaller lesion size (*p *< 0.01, Fig. 4D).
E. Treatment start after SCI: More than two thirds of the interventions were applied before or immediately after the injury. The largest effect size was observed when the intervention has been started within 14 days after injury. Subsequent treatment resulted in a reduction of effect sizes (*p *< 0.01, Fig. 4E).
F. Follow-up (observational) time window: When animal experiments were assessed within one month after SCI, the effect sizes were higher when compared to experiments assessed up to nine months after SCI (*p *< 0.01, Fig. 4F).
G. The number of animals in the treatment group had a significant inverse effect on reported efficacy. Groups with 7 or less animals demonstrated between twice and three times the effect size compared to larger animal groups (*p *< 0.01, Fig. 4G).
H. Item randomisation accounted for a significant amount of between-study heterogeneity as the only item of the quality score. Randomised studies provided significant benefit (15.5%), but this was 8.9% lower than reported by non-randomised studies (*p *= 0.03, Fig. 4H). Of note, blinded assessment of outcome revealed an effect size of 17.9 (95% CI 13.4–22.4) in 34 experiments whereas studies without blinding showed an effect size of 23.7 (95% CI 6.7–40.6). However, blinded assessment of outcome did not account for a statistically significant amount of heterogeneity (*p *= 0.41, data not shown).

### Evaluation of theoretical missing data

We assessed the obtained data-sets for potentially underlying (publication) bias applying funnel plotting and Egger regression analysis for small study effects. Publication bias is a major source of missing data and attributed to unpublished studies. Funnel plotting for the 40 included experiments reported an overall neurobehavioral efficacy of 18.7% (95% CI 14.4–23.0). Several methods were applied to assess for missing data (Fig. 5A). Fist, trim and fill analysis imputed 19 missing experiments resulting in a reduced effect size of 8.8% (95% CI 2.6–14.9) (Fig. 5B). Second, Egger regression detected an intercept of 2.34 suggestive of missing data and confirmed statistical significancy in the egger test (*p *< 0.005, Supplemental Fig. 1). Third, contour-enhanced funnel plot verified the existence of publication bias (Fig. 5C). The majority of experiments were located close to the most common limit of statistical significance (*p* value of 0.05) or within the area of high statistical significance (*p *< 0.01), whereas less than one fourth of experiments were located in the area of statistical non-significance^40^.

## Discussion

### Overall study results

This systematic review and meta-analysis of preclinical SCI experiments includes data of 1572 animals reporting inhibition of the Nogo-A pathway after traumatic spinal cord injury. Systematic reviews and meta-analyses can provide empirical evidence of preclinical interventions^41^. Among the main options to intervene with Nogo pathway, the application of NgR blockers revealed the highest effect sizes followed by Nogo-A antibody interventions. This may be due to capacity of NgR-based interventions to block several inhibitory proteins in addition to Nogo-A. The effect of Nogo-A and NgR loss of function (genetic) knockouts have been lower possibly indicating compensatory effects such as elevated levels of semaphorins and ephrins and their receptors in the CNS of mice lacking Nogo‑A, as described before^42^. Another underlying reason could be the variable sequences being knocked out in the Nogo-A or NgR gene domain which are coding for different proteins with variable functional relevance for impeding outgrowth. Another possibility is that compared to genetic deletion of Nogo-A/NgR protein-based interventions through Nogo-A antibodies or Nogo-Receptor blockers may cause further protein–protein interactions interfering with the downstream signalling beyond NgR-1 only.

### Subgroup analysis to reduce Heterogeneity

After screening for sources of heterogeneity attributed to differences in SCI modelling, we identified experimental SCI in rats using BBB locomotor assessments as the being the largest (N = 890) and most homogenous group (smallest CI). To reduce heterogeneity the subsequent analysis was characterized by the use of the same neurobehavioral scores and animal model. Here an overall effect size of 18.7% was calculated.

In the following we summarized variable effects sizes attributable to variable aspects of SCI modelling. Substantial differences in neurobehavioral outcome were revealed based on the method of SCI induction (Fig. 4). Complete injuries such as compression or transection of the spinal cord showed the lowest efficacy for Nogo-A pathway inhibition, whereas incomplete lesions such as dorsal and lateral hemisections revealed double the effect size than the other.

Sprouting and plasticity of spared axons after incomplete SCI could be improved by certain treatments when there are sufficient spared axons, but that these same treatments might on their own not mediate axon regeneration across complete lesions and therefore do not have any effect on recovery in cases of severe SCI with no or few spared axons. Beneficial inhibition of the Nogo-A pathway might only occur within incomplete injuries as demonstrated in previous studies^36^. Furthermore, differences in hemisection injuries have been revealed in the subgroup analysis where lateral hemisections almost reported double the effect size compared to dorsal hemisection models. The anatomy of the spinal cord concludes in partially spontaneous recovery of hindlimb stepping for unilaterally injured animals making the evaluation of any treatment to laterally hemisected animals problematic. Cervical injuries resulted in neurobehavioral outcome with immense variability pointing toward a lack of a uniformly accepted standardized cervical injury model (Fig. 4). Thoracic injuries are most prevalent, predominantly localized from T8 to T10 and demonstrated similar effect sizes. Impactor-induced contusion injuries resulted in smaller effect sizes of motor recovery compared to partial transection injuries (Fig. 4B,C), as being reported earlier^37,43^.

Most of the included studies applied treatments before or immediately after SCI associated with neurobehavioral outcome higher than the overall estimate of efficacy (Fig. 4E). However, experiments with intervention starting seven days after SCI reported the highest effect size within this stratification. A delayed treatment start at more than 14 days post SCI was associated with a significantly reduced effect size. The follow-up time window allows to estimate for the ‘durability of effects’ after SCI. Over the first month the effects are highest and subside thereafter, possibly pointing towards the accumulating emergence of negative outcome modifier, such as acquired infections. In large clinical SCI studies, acquired infections have been identified earlier as independent risk factor for neuroworsening^44^. The observation that effect sizes diminish with extended observational period has been detected in other studies, irrespective of the specific intervention^37^. Hence this effect is likely also independent from the specific intervention investigated here.

### Small sample sizes and study quality

Small sample sizes in animal experiments are prone to distort the effect sizes if animals are lost during the experiments (attrition bias)^45^. We observed a relative exaggeration of efficacy for animal studies reporting the use of less than 8 animals in the treatment group (Fig. 4F). Additionally, the reporting of randomization in the published studies reduced the reported effect size by 37%. Missing data is suggested by asymmetrical funnel plots and confirmed by Egger-regression analysis (Fig. 5). Factoring in predicted missing experiments by Trim and fill analysis resulted in a reduced effect size by 53%. However, the quantitative reduction of efficacy from 18.7% to 8.8% after the imputation of the trim-and-fill method has to be appraised critically; an effect size of 8.8% would only represent 1–2 points on the 21-points BBB score. As discussed in the literature before, adjusted intervention effect estimates from the trim-and-fill method alone should be interpreted with caution since the method is limited in the presence of substantial between-study heterogeneity^46^ (see also 4.4.).

### Limitations

The applied statistical methods in our study are detailed, provide reliable results and are most commonly used within meta-analysis, but they are also intensively discussed and questioned within the statistical community. Four different approaches were used to detect potentially underlying (publication) bias within this study (funnel plotting, the trim-and-fill method, contour-enhanced funnel plot and egger-regression) to address this matter. However, we emphasize the careful interpretation of deductions from single statistical methods, e.g. the funnel plot asymmetry alone. Within funnel plots the distribution of effect sizes is caused by the studies’ variance, but does not account for possible different true effects^47^ based on study characteristics such as different method of SCI induction or different animal models. Another limitation is that different outcome testing modalities need to be chosen carefully with respect to the specific injury model, severity, and location^48^. Our analysis cannot discriminate whether the chosen surrogate outcome parameter BBB was truly justified to detect the best possible effect in all published experiments. Moreover, similar percentage changes measured by different scores may refer to different meanings of terms of functional relevance. Regarding the used neurobehavioral scores, the accuracy of our analysis relies on the reliability of the used locomotor recovery scales (e.g. BBB score). The standard BBB score constitutes a 21 item score and small differences of a point or two might be recorded as statistically significant but actually be functionally meaningless in certain parts of the scale. This limitation has been discussed in the literature and transformations of the BBB score has been proposed^49^ based on the underlying raw data which is not available in this meta-analyis. On the other side, these scales to measure outcomes are extensively used and recognized as the most informative and commonly used neurobehavioral tool to evaluate the results of spinal cord injury models. Therefore, it's unlikely for a preclinical intervention to be regarded for translation if it doesn't lead to enhancements in the BBB score.

### Conclusion

This systematic review and meta-analysis suggest an effect of Nogo-A pathway inhibition on improving neurological recovery after certain models of experimental SCI. Subgroup analysis suggest that this effect diminished with time after SCI. In line with other interventions tested in experimental SCI^37^ we observe similar factors associated with outcome heterogeneity.

## Material and methods

### Systematic database search

To identify studies describing the effect of inhibition of the Nogo-A pathway after SCI we conducted an electronical search in PubMed, EMBASE and ISI Web of Science, using the following search term.

(”Nogo” OR “Nogo-66” OR “Nogo-A” OR “RTN4a” OR “Anti-Nogo” OR “NgR” OR “Nogo-Receptor” OR “NgR1” OR “NgR2” OR “MAG” OR “Omgp” OR “LRP1” OR “TNFR” OR “p75” OR “TROY” OR “Lingo-1” OR “PirB” OR “In-1” OR “NEP1-40” OR “Nogo antibody” OR “Nogo antibodies” OR “neurite growth inhibitors” OR “myelin inhibitors“) AND (“spinal cord injury” OR “hemisection” OR “contusion” OR “dorsal column injury” OR “transection” OR “corticospinal tract injury” OR “compression” OR “spinal cord lesion”). The search results were filtered for experiments using animals if a filter was available. We used a modified animal filter for the search in Pubmed^50^. The initial search was performed on March 30, 2015 and updated on March 04, 2022 based on a study protocol finalized in advance of any data collection (accessible online https://syrf.org.uk/protocols).

### Inclusion and exclusion criteria

Studies were included when they reported the effect of Nogo-A pathway inhibition in *in-vivo* models of traumatic animal spinal cord injury assessing motor recovery. Inhibition of the Nogo-A pathway was targeted by various therapeutic strategies: application of inhibitory drugs (e.g. antibodies, peptides, enzymes), knock-out models (KO), vaccination against receptors and signalling molecules, suppression of receptor expression or signalling molecules via lentivirally carried small hairpin RNA (siRNA). There were no restrictions on the published time period, the animal model or reporting language. Full publications as well as conference abstracts were included. Non-traumatic models of SCI and studies which reported only synergistic effects of combined treatment were excluded. Studies had to report the number of animals in the control and treatment group, the mean effect size and its variance (standard deviation or standard error of the mean).

### Data extraction

Study characteristics which were extracted included the gender and breed of the animals, time, route and dose of application, anaesthetic used, method of injury induction and additional treatment. Functional outcome was assessed for each experiment comparing the group of animals receiving the treatment and control. Where the outcome was expressed graphically only, Universal Desktop Ruler (Version 3.6, AVPsoft) was used to visually extract the data points. Where data was expressed serially, numerical values were extracted. Only the final time point of the assessment of motor recovery was included for each group of animals.

### Quality assessment

The methodological quality of each study was assessed using a modified 9-point item quality checklist, adapted from the CAMARADES (Collaborative Approach to Meta-Analysis and Review of Animal Data from Experimental Studies) quality checklist^51^: (i) reporting of a sample size calculation, (ii) control of animals’ temperature, (iii) use of anaesthetics other than ketamine (because of its previously reported intrinsic neuroprotective activity), (iv) randomised treatment allocation, (v) treatment allocation concealment, (vi) blinded assessment of outcome, (vii) publication in a peer reviewed journal; (viii) statement of compliance with regulatory requirements and (ix) statement of potential conflicts of interest.

### Analysis

A normalized effect size (ES) was calculated for each comparison which is defined as the improvement of outcome in the treatment group compared to the control group adjusted for the maximum outcome in the motor score (normally represented by sham animals undergoing laminectomy surgery without injury to the spinal cord). The size of the control group was adjusted if a single control group was compared to more than one treatment group.

We used random effects meta-analysis to calculate an overall estimate of effect size. The analysis was stratified according to the method of injury induction, type of SCI (complete / incomplete), type of treatment, time of treatment application, quality assessment score, additional treatment, time of assessment, year of publication, details of surgical procedure and type of anaesthetic. The *metafor*-package of R was used for meta-analysis^52^, the significance level was set at *p *= 0.05. To assess how much residual heterogeneity was explained by each independent variable within a stratified dataset adjusted R^2^ values were calculated^53^. Restricted maximum likelihood (REML) was used to estimate the additive (between-study) component of variance τ^2^ within the meta-analysis and the Hartung-Knapp adjustment for random effects models^52^. The dependent variable was the normalized effect size in all cases. The method and statistical approach is described in greater detail elsewhere^53^. Funnel plots and trim and fill analyses^54^ were performed by plotting normalized effect size against precision (1/standard error of the mean) using the R package *meta*. Egger regression (*meta*-package in R) is used to detect the possible presence of publication bias measured by the intercept from standard normal deviates against precision^55^. Contour-enhanced funnel plot was performed using the R package *meta*.

### Supplementary Information

Supplementary Information.

## Data Availability

A study protocol was finalized in advance of any data collection. The study protocol is registered at the systematic review facility: https://syrf.org.uk/protocols. The analysis plan is included in the study protocol. The analysis was conducted according to the plan. Study selection is accounting for all experimental subjects is included within the figures according to the PRISMA guidelines for flow diagrams. Data from this study will be made available (as allowable according to institutional regulatory standards) by e-mailing the corresponding author. Analytic code used to conduct the analyses presented in this study are not available in a public repository. It can be made available upon request to the corresponding author. The authors agree or have agreed to publish the manuscript using “Open Access” option under appropriate license.
