# Supplementary material for: Inhibition of the Nogo-pathway in experimental spinal cord injury: a meta-analysis of 76 experimental treatments
Source: Sci Rep. 2023 Dec 21;13:22898. doi: 10.1038/s41598-023-49260-5 (PMC10739940; doi:10.1038/s41598-023-49260-5)

SUPPLEMENT

Inhibition of the Nogo-pathway in experimental spinal cord injury – a meta-analysis

Supplemental Table 1: Details of included studies

Study	Drug	Score used	No. of animals	Animal type	Lesion level	Timepoint of administra-tion (hours)	Time of assessment (days)	Anaesthetic	Type of injury (Complete / Incomplete)	Outcome values (intervention | control)
Atalay (Atalay et al., 2007)	Anti-NOGO/NEP1-40	Others	15	Rat	Th10	0	14-21	Ketamine and xylazine	Lateral Hemisection (Incomplete)	3.75 | 0.86
Bregman (Bregman et al., 1995)	Anti-NOGO-A	Others	20	Rat	Th6-9	168	22-28	Unknown	Dorsal Hemisection (Incomplete)	164.71 | 145.88
Cafferty (Cafferty et al., 2010)	Nogo knockout	BMS	25	Mouse	Th6	5	22-28	Ketamine and xylazine	Dorsal Hemisection (Incomplete)	4.75 | 3.37
Cao (Cao et al., 2008)	Anti-NOGO/NEP1-40	BBB	13	Rat	C3	168	56	Acepromacine, ketamin, xylazine	Lateral Hemisection (Incomplete)	16.64 | 15.34
Cen (Cen et al., 2013)	Anti-NgR1	BBB	20	Rat	Th10	72	56	Chloral Hydrate	Transection (Complete)	8.40 | 2.36
Donaghue (Elliott Donaghue et al., 2016)	Anti-NOGO-A	BBB	28	Rat	Th2	0	56	Isoflurane	Compression 26g (complete)	10.65 | 10.16
Freund (Freund et al., 2009)	Anti-NOGO-A	Others	12	Monkey	C7-8	4	89 - 280	Ketamine & Pentobarbital	Lateral Hemisection (Incomplete)	83.5 | 53.83
Geoffroy (Geoffroy et al., 2015)	Nogo knockout	BMS / Others	42	Mouse	Th8	-1008	42	Avertin	Dorsal Hemisection (Incomplete)	(1) 5.22 | 5.78<br>(2) 74.52 | 77.08<br>(3) 30.91 | 50.16
Gonzenbach (Gonzenbach et al., 2012)	Anti-NOGO-A	Others	16	Rat	T8	0	63 - 70	Hypnorm and Dormicum	Dorsal Hemisection (Incomplete)	(1) 4.57 | 2.44<br>(2) 4.3 | 2.55<br>(3) 4.3 | 5.26
GrandPre (GrandPre et al., 2002)	Anti-NOGO/NEP1-40	BBB	13	Rat	Th6	72	22-28	Ketamine and xylazine	Dorsal Hemisection (Incomplete)	15.52 | 11.49
Harel (Harel et al., 2010)	Nogo knockout	BMS	31	Mouse	C3 - C4	168	89 - 280	Ketamine and xylazine	Lateral Hemisection	6.83 | 7.18

									(Incomplete)	
Hauben (Hauben et al., 2001)	Anti-NOGO-A	BBB	12	Rat	T9	0	89 - 280	Ketamine and xylazine	Contusion 10g (Incomplete)	8 | 3.01
Hirokawa (Hirokawa et al., 2017)	Nogo knockout	BMS / Others	58	Mouse	Th8	-1008	28	Isoflurane	Dorsal Hemisection (Incomplete)	(1) 5.85 | 3.41 (2) 5.20 (3.41)
Ito (Ito et al., 2018)	Nogo knockout	BMS	10	Mouse	Th10	-1008	42	Ketamine and Xylazine	Contusion 70g (complete)	4.13 | 2.27
Ji (Ji et al., 2005)	Anti-NOGO-A	BBB	27	Rat	Th6	2016	22-28	Midazolam + Halothane	Dorsal Hemisection (Incomplete)	14.8 | 12
Ji (Ji et al., 2006)	Anti-NgR1	BBB	15	Rat	Th7	-672	22-28	Ketamine and xylazine	Dorsal Hemisection (Incomplete)	13.1 | 10.8
Ji (Ji et al., 2008)	Nogo knockout	BMS	28	Mouse	Th10	0	35-49	Ketamine and xylazine	Dorsal Hemisection (Incomplete)	1.53 | 0.87
Kim (Kim et al., 2003)	Nogo knockout	BBB	39	Mouse	Th6	0	14-21	Ketamine and xylazine	Dorsal Hemisection (Incomplete)	13.62 | 10.78
Kim (Kim et al., 2004)	Nogo knockout	BBB	20	Mouse	Th6	0	14-21	Ketamine and xylazine	Dorsal Hemisection (Incomplete)	13.66 | 9.77
Lan (Lan and Song, 2009)	Anti-NOGO-A	BBB	18	Rat	Th8-9	0	63 - 70	Chloral Hydrate	Transection (Complete)	(1) 7.44 | 7.56 (2) 8.89 | 7.56
Li (Li and Strittmatter, 2003)	Anti-NOGO/NEP1-40	BBB	16	Mouse	Th6	0	35-49	Ketamine and xylazine	Dorsal Hemisection (Incomplete)	(1) 15.62 | 13.15 (2) 15.7 | 13.22 (3) 13.83 | 9.48
Li (Li et al., 2004)	Anti-NOGO-A	BBB	50	Rat	Th6	168	22-28	Ketamine and xylazine	Dorsal Hemisection (Incomplete)	(1) 15.61 | 12.78 (2) 13.1 | 10.04 (3) 13.85 | 12.13
Li (Li et al., 2005)	Anti-NOGO-A	BBB	11.5	Mouse	Th6	0	22-28	Ketamine and xylazine	Dorsal Hemisection (Incomplete)	(1) 15.73 | 12.34 (2) 16.24 | 12.34
Liu (Liu et al., 2016)	Anti-NOGO-A	BBB	8	Rat	Th10	168	10	Chloral Hydrate	Lateral Hemisection (incomplete)	12.61 | 5.11
Lu (Lu et al., 2010)	Anti-NgR1	BBB	24	Rat	Th8	0	56	Sodium Pentobarbital	Contusion (Incomplete)	15.7 | 13.7
Lu (Lu et al., 2019)	NgR1-immunisation/siRNA	BBB	18	Rat	Th10	-1008	42	Sodium Pentobarbital	Dorsal Hemisection	(1) 16.65 | 13.67 (2) 18.56 | 13.67

									(Incomplete)	
Lv (Lv et al., 2010)	Anti-NOGO-A	BBB	31	Rat	Th9	-1008	22-28	Ketamine and xylazine	Dorsal Hemisection (Incomplete)	14.39 | 12.09
Lv (Lv et al., 2012)	Anti-NgR1	BBB	33	Rat	Th8	0	63 - 70	Sodium Pentobarbital	Contusion 10g (Incomplete)	15.69 | 13.68
Merkler (Merkler et al., 2001)	Anti-NOGO-A	BBB	34	Rat	Th8	0	35-49	Fentanyl+Midazolam	Dorsal Hemisection (Incomplete)	12.5 | 10.3
Nakamura (Nakamura et al., 2011)	Anti-NOGO/NEP1-40	BMS	14	Mouse	Th9	336	35-49	Sodium Pentobarbital	Dorsal Hemisection (Incomplete)	(1) 3.43 | 3.94<br>(2) 3.64 | 3.86<br>(3) 4.05 | 3.86
Steward (Steward et al., 2008)	Anti-NOGO/NEP1-40	BBB	14	Mouse	Th6	0	22-28	Avertin	Dorsal Hemisection (Incomplete)	(1) 5.14 | 4.62<br>(2) 5.24 | 5.07<br>(3) 6.1 | 5.38
Tong (Tong et al., 2014)	Nogo knockout	BMS	6	Mouse	Th8	0	35-49	Ketamine and Xylazine	Dorsal Hemisection (Incomplete)	5.93 | 6.99
Wang (Wang et al., 2006)	Anti-NOGO-A	BBB	16	Rat	Th8	0	35-49	Ketamine and xylazine	Contusion 10g (Incomplete)	(1) 10.44 | 9.25<br>(2) 9.5 | 6.75
Wang (Wang et al., 2011a)	Anti-NOGO-A	BBB	64	Rat	Th7	0	89 - 280	Ketamine and xylazine	(1) Dorsal Hemisection<br>(2) Contusion 10g (Incomplete	(1) 4.25 | 3.4<br>(2) 8.65 | 8.1
Wang (Wang et al., 2011b)	Anti-NgR1	BBB	9	Rat	Th9	0	35-49	Pentobarbital	Dorsal Hemisection	19.64 | 17.55
Wang (Wang et al., 2014)	Anti-NOGO-A	BBB	16	Rat	Th7	168	63 - 70	Ketamine and Xylazine	Contusion	(1) 8.01 | 7.98<br>(2) 9.52 | 8.33<br>(3) 9.75 | 8.33<br>(4) 10.05 | 8.13
Wang (Wang et al., 2015)	Anti-p75-Ab	BBB	24	Rat	Th10	0	42	Sodium Pentobarbital	Dorsal Hemisection (Incomplete)	(1) 18.61 | 17.87<br>(2) 3.47 | 3.74)
Wang (Wang et al., 2020)	Anti-NgR1	Others	13	Monkey	C5	720	180	Isoflurane	Lateral Hemisection (Incomplete)	(1) 0.17 | 0.05<br>(2) 3.72 | 2.18<br>(3) 2.56 | 1.83
Wu (Wu et al., 2013)	Anti-NgR1	BBB	20	Rat	Th10	0	56	Chloral Hydrate	Transection (Complete)	7.29 | 2.30
Wu (Wu et al., 2020)	Anti-LINGO-1	BBB	48	Rat	Th10	0	56	Unknown	Transection (Complete)	8.50 | 2.70

Xu (Xu et al., 2004)	Anti-NgR1	BBB	12	Rat	Th8-9	0	22-28	Ketamine and Xylazine	Dorsal Hemisection (Incomplete)	19.6 | 16.2
Xu (Xu et al., 2011)	Anti-NgR1	BBB	19	Rat	Th9	0	89 - 280	Pentobarbital Sodium	Contusion 10g (Incomplete)	13.57 | 11.4
Yan (Yan et al., 2009)	Anti-NOGO-A	BBB	20	Rat	Th10	72	56	Unknown	Transection (Complete)	7.89 | 7.7
Yu (Yu et al., 2007)	Anti-NgR1	BBB	18	Rat	Th9	168	56	Pentobarbital	Contusion (10g) (Incomplete)	15.2 | 13.42
Yu (Yu et al., 2008)	Anti-NgR1	BBB	8	Rat	Th9	0	56	Pentobarbital Sodium	Contusion 10g (incomplete)	15.35 | 13.3
Zhan (Zhan et al., 2008)	Anti-NOGO-A	BBB	12	Rat	Th8	-1344	22-28	Chloral Hydrate	Dorsal Hemisection (incomplete)	13.92 | 10.72
Zhang (Zhang et al., 2009)	Anti-NOGO-A	BBB	40	Rat	Th8	0	14-21	Pentobarbital Sodium	Dorsal Hemisection (Incomplete)	14 | 12
Zhang (Zhang et al., 2013)	Anti-NOGO-A	BBB	16	Rat	Th10	72	63 - 70	Unknown	Transection (Complete)	8.89 | 7.46
Zhao (Zhao et al., 2018)	NgR1-immunisation/siRNA	BBB	52	Rat	Th10	0	56	Chloral Hydrate + Ketamine	Transection (Complete)	8.57 | 5.48
Zheng (Zheng et al., 2003)	Nogo knockout	BBB	22	Mouse	Th7	0	14-21	Avertin	Dorsal Hemisection (Incomplete)	(1) 14 | 12.62<br>(2) 10.70 | 11.12
Zhilai (Zhilai et al., 2011)	Anti-NOGO/NEP1-40	BBB	16	Rat	Th10	0	63 - 70	Sodium Pentobarbital	Contusion (Incomplete)	9.35 | 9.08

Atalay, B., Bavbek, M., Cekinmez, M., Ozen, O., Nacar, A., Karabay, G., Gulsen, S., 2007. Antibodies neutralizing Nogo-A increase pan-cadherin expression and motor recovery following spinal cord injury in rats. Spinal cord 45, 780-786.

Bregman, B.S., Kunkelbagden, E., Schnell, L., Dai, H.N., Gao, D., Schwab, M.E., 1995. Recovery from Spinal-Cord Injury Mediated by Antibodies to Neurite Growth-Inhibitors. Nature 378, 498-501.

Cafferty, W.B., Duffy, P., Huebner, E., Strittmatter, S.M., 2010. MAG and OMgp synergize with Nogo-A to restrict axonal growth and neurological recovery after spinal cord trauma. The Journal of neuroscience : the official journal of the Society for Neuroscience 30, 6825-6837.

Cao, Y., Shumsky, J.S., Sabol, M.A., Kushner, R.A., Strittmatter, S., Hamers, F.P., Lee, D.H., Rabacchi, S.A., Murray, M., 2008. Nogo-66 receptor antagonist peptide (NEP1-40) administration promotes functional recovery and axonal growth after lateral funiculus injury in the adult rat. Neurorehabilitation and neural repair 22, 262-278.

Cen, J., Wu, H., Ren, X., Zhang, H., Wang, J., Wan, Y., Deng, Y., 2013. Local injection of lentivirus encoding LINGO-1-shRNA promotes functional recovery in rats with complete spinal cord transection. Spine 38, 1632-1639.

Elliott Donaghue, I., Tator, C.H., Shoichet, M.S., 2016. Local Delivery of Neurotrophin-3 and Anti-NogoA Promotes Repair After Spinal Cord Injury. Tissue engineering. Part A 22, 733-741.

Freund, P., Schmidlin, E., Wannier, T., Bloch, J., Mir, A., Schwab, M.E., Rouiller, E.M., 2009. Anti-Nogo-A antibody treatment promotes recovery of manual dexterity after unilateral cervical lesion in adult primates - re-examination and extension of behavioral data. European Journal of Neuroscience 29, 983-996.

Geoffroy, C.G., Lorenzana, A.O., Kwan, J.P., Lin, K., Ghassemi, O., Ma, A., Xu, N., Creger, D., Liu, K., He, Z., Zheng, B., 2015. Effects of PTEN and Nogo Codeletion on Corticospinal Axon Sprouting and Regeneration in Mice. The Journal of neuroscience : the official journal of the Society for Neuroscience 35, 6413-6428.

Gonzenbach, R.R., Zoerner, B., Schnell, L., Weinmann, O., Mir, A.K., Schwab, M.E., 2012. Delayed anti-nogo-a antibody application after spinal cord injury shows progressive loss of responsiveness. Journal of neurotrauma 29, 567-578.

GrandPre, T., Li, S., Strittmatter, S.M., 2002. Nogo-66 receptor antagonist peptide promotes axonal regeneration. Nature 417, 547-551.

Harel, N.Y., Song, K.H., Tang, X., Strittmatter, S.M., 2010. Nogo receptor deletion and multimodal exercise improve distinct aspects of recovery in cervical spinal cord injury. Journal of neurotrauma 27, 2055-2066.

Hauben, E., Ibarra, A., Mizrahi, T., Barouch, R., Agranov, E., Schwartz, M., 2001. Vaccination with a Nogo-A-derived peptide after incomplete spinal-cord injury promotes recovery via a T-cell-mediated neuroprotective response: comparison with other myelin antigens. Proceedings of the National Academy of Sciences of the United States of America 98, 15173-15178.

Hirokawa, T., Zou, Y., Kurihara, Y., Jiang, Z., Sakakibara, Y., Ito, H., Funakoshi, K., Kawahara, N., Goshima, Y., Strittmatter, S.M., Takei, K., 2017. Regulation of axonal regeneration by the level of function of the endogenous Nogo receptor antagonist LOTUS. Scientific reports 7, 12119.

Ito, S., Nagoshi, N., Tsuji, O., Shibata, S., Shinozaki, M., Kawabata, S., Kojima, K., Yasutake, K., Hirokawa, T., Matsumoto, M., Takei, K., Nakamura, M., Okano, H., 2018. LOTUS Inhibits Neuronal Apoptosis and Promotes Tract Regeneration in Contusive Spinal Cord Injury Model Mice. eNeuro 5.

Ji, B., Case, L.C., Liu, K., Shao, Z., Lee, X., Yang, Z., Wang, J., Tian, T., Shulga-Morskaya, S., Scott, M., He, Z., Relton, J.K., Mi, S., 2008. Assessment of functional recovery and axonal sprouting in oligodendrocyte-myelin glycoprotein (OMgp) null mice after spinal cord injury. Molecular and cellular neurosciences 39, 258-267.

Ji, B., Li, M., Budel, S., Pepinsky, R.B., Walus, L., Engber, T.M., Strittmatter, S.M., Relton, J.K., 2005. Effect of combined treatment with methylprednisolone and soluble Nogo-66 receptor after rat spinal cord injury. The European journal of neuroscience 22, 587-594.

Ji, B., Li, M., Wu, W.T., Yick, L.W., Lee, X., Shao, Z., Wang, J., So, K.F., McCoy, J.M., Blake Pepinsky, R., Mi, S., Relton, J.K., 2006. LINGO-1 antagonist promotes functional recovery and axonal sprouting after spinal cord injury. Molecular and Cellular Neuroscience 33, 311-320.

Kim, J.E., Li, S., GrandPre, T., Qiu, D., Strittmatter, S.M., 2003. Axon regeneration in young adult mice lacking Nogo-A/B. Neuron 38, 187-199.

Kim, J.E., Liu, B.P., Park, J.H., Strittmatter, S.M., 2004. Nogo-66 receptor prevents raphespinal and rubrospinal axon regeneration and limits functional recovery from spinal cord injury. Neuron 44, 439-451.

Lan, H., Song, Y., 2009. Effects of poly lactic-co-glycolic acid-Nogo A antibody delayed-release microspheres on regeneration of injured spinal cord in rats. Neural Regeneration Research 4, 358-364.

Li, S., Kim, J.E., Budel, S., Hampton, T.G., Strittmatter, S.M., 2005. Transgenic inhibition of Nogo-66 receptor function allows axonal sprouting and improved locomotion after spinal injury. Molecular and cellular neurosciences 29, 26-39.

Li, S., Liu, B.P., Budel, S., Li, M., Ji, B., Walus, L., Li, W., Jirik, A., Rabacchi, S., Choi, E., Worley, D., Sah, D.W., Pepinsky, B., Lee, D., Relton, J., Strittmatter, S.M., 2004. Blockade of Nogo-66, myelin-associated glycoprotein, and oligodendrocyte myelin glycoprotein by soluble Nogo-66 receptor promotes axonal sprouting and recovery after spinal injury. The Journal of neuroscience : the official journal of the Society for Neuroscience 24, 10511-10520.

Li, S., Strittmatter, S.M., 2003. Delayed systemic Nogo-66 receptor antagonist promotes recovery from spinal cord injury. The Journal of neuroscience : the official journal of the Society for Neuroscience 23, 4219-4227.

Liu, G.M., Luo, Y.G., Li, J., Xu, K., 2016. Knockdown of Nogo gene by short hairpin RNA interference promotes functional recovery of spinal cord injury in a rat model. Molecular medicine reports 13, 4431-4436.

Lu, B.T., Yuan, W., Xu, S.M., 2010. [Lentiviral vector-mediated RNA interfere gene Nogo receptor to repair spinal cord injury]. Zhonghua wai ke za zhi [Chinese journal of surgery] 48, 1573-1576.

Lu, X.M., Mao, M., Xiao, L., Yu, Y., He, M., Zhao, G.Y., Tang, J.J., Feng, S., Li, S., He, C.M., Wang, Y.T., 2019. Nucleic Acid Vaccine Targeting Nogo-66 Receptor and Paired Immunoglobulin-Like Receptor B as an Immunotherapy Strategy for Spinal Cord Injury in Rats. Neurotherapeutics : the journal of the American Society for Experimental NeuroTherapeutics.

Lv, B., Yuan, W., Xu, S., Zhang, T., Liu, B., 2012. Lentivirus-siNgR199 promotes axonal regeneration and functional recovery in rats. The International journal of neuroscience 122, 133-139.

Lv, J., Xu, R.X., Jiang, X.D., Lu, X., Ke, Y.Q., Cai, Y.Q., Du, M.X., Hu, C., Zou, Y.X., Qin, L.S., Zeng, Y.J., 2010. Passive immunization with LINGO-1 polyclonal antiserum afforded neuroprotection and promoted functional recovery in a rat model of spinal cord injury. Neuroimmunomodulation 17, 270-278.

Merkler, D., Metz, G.A., Raineteau, O., Dietz, V., Schwab, M.E., Fouad, K., 2001. Locomotor recovery in spinal cord-injured rats treated with an antibody neutralizing the myelin-associated neurite growth inhibitor Nogo-A. The Journal of neuroscience : the official journal of the Society for Neuroscience 21, 3665-3673.

Nakamura, Y., Fujita, Y., Ueno, M., Takai, T., Yamashita, T., 2011. Paired immunoglobulin-like receptor B knockout does not enhance axonal regeneration or locomotor recovery after spinal cord injury. The Journal of biological chemistry 286, 1876-1883.

Steward, O., Sharp, K., Yee, K.M., Hofstadter, M., 2008. A re-assessment of the effects of a Nogo-66 receptor antagonist on regenerative growth of axons and locomotor recovery after spinal cord injury in mice. Experimental neurology 209, 446-468.

Tong, J., Ren, Y., Wang, X., Dimopoulos, V.G., Kesler, H.N., Liu, W., He, X., Nedergaard, M., Huang, J.H., 2014. Assessment of Nogo-66 receptor 1 function in vivo after spinal cord injury. Neurosurgery 75, 51-60.

Wang, X., Baughman, K.W., Basso, D.M., Strittmatter, S.M., 2006. Delayed Nogo receptor therapy improves recovery from spinal cord contusion. Annals of neurology 60, 540-549.

Wang, X., Duffy, P., McGee, A.W., Hasan, O., Gould, G., Tu, N., Harel, N.Y., Huang, Y., Carson, R.E., Weinzimmer, D., Ropchan, J., Benowitz, L.I., Cafferty, W.B., Strittmatter, S.M., 2011a. Recovery from chronic spinal cord contusion after Nogo receptor intervention. Annals of neurology 70, 805-821.

Wang, X., Yigitkanli, K., Kim, C.Y., Sekine-Komo, T., Wirak, D., Frieden, E., Bhargava, A., Maynard, G., Cafferty, W.B., Strittmatter, S.M., 2014. Human NgR-Fc decoy protein via lumbar intrathecal bolus administration enhances recovery from rat spinal cord contusion. Journal of neurotrauma 31, 1955-1966.

Wang, X., Zhou, T., Maynard, G.D., Terse, P.S., Cafferty, W.B., Kocsis, J.D., Strittmatter, S.M., 2020. Nogo receptor decoy promotes recovery and corticospinal growth in non-human primate spinal cord injury. Brain : a journal of neurology 143, 1697-1713.

Wang, Y.T., Lu, X.M., Zhu, F., Huang, P., Yu, Y., Long, Z.Y., Wu, Y.M., 2015. Ameliorative Effects of p75NTR-ED-Fc on Axonal Regeneration and Functional Recovery in Spinal Cord-Injured Rats. Molecular neurobiology 52, 1821-1834.

Wang, Y.T., Lu, X.M., Zhu, F., Huang, P., Yu, Y., Zeng, L., Long, Z.Y., Wu, Y.M., 2011b. The use of a gold nanoparticle-based adjuvant to improve the therapeutic efficacy of hNgR-Fc protein immunization in spinal cord-injured rats. Biomaterials 32, 7988-7998.

Wu, H., Ding, L., Wang, Y., Zou, T.B., Wang, T., Fu, W., Lin, Y., Zhang, X., Chen, K., Lei, Y., Zhong, C., Luo, C., 2020. MiR-615 Regulates NSC Differentiation In Vitro and Contributes to Spinal Cord Injury Repair by Targeting LINGO-1. Molecular neurobiology 57, 3057-3074.

Wu, H.F., Cen, J.S., Zhong, Q., Chen, L., Wang, J., Deng, D.Y., Wan, Y., 2013. The promotion of functional recovery and nerve regeneration after spinal cord injury by lentiviral vectors encoding Lingo-1 shRNA delivered by Pluronic F-127. Biomaterials 34, 1686-1700.

Xu, C.J., Xu, L., Huang, L.D., Li, Y., Yu, P.P., Hang, Q., Xu, X.M., Lu, P.H., 2011. Combined NgR vaccination and neural stem cell transplantation promote functional recovery after spinal cord injury in adult rats. Neuropathology and applied neurobiology 37, 135-155.

Xu, G., Nie, D.Y., Chen, J.T., Wang, C.Y., Yu, F.G., Sun, L., Luo, X.G., Ahmed, S., David, S., Xiao, Z.C., 2004. Recombinant DNA vaccine encoding multiple domains related to inhibition of neurite outgrowth: a potential strategy for axonal regeneration. Journal of neurochemistry 91, 1018-1023.

Yan, H.B., Zhang, Z.M., Jin, D.D., Wang, X.J., Lu, K.W., 2009. [The repair of acute spinal cord injury in rats by olfactory ensheathing cells graft modified by glia cell line-derived neurotrophic factor gene in combination with the injection of monoclonal antibody IN-1]. Zhonghua wai ke za zhi [Chinese journal of surgery] 47, 1817-1820.

Yu, P., Huang, L., Zou, J., Yu, Z., Wang, Y., Wang, X., Xu, L., Liu, X., Xu, X.M., Lu, P.H., 2008. Immunization with recombinant Nogo-66 receptor (NgR) promotes axonal regeneration and recovery of function after spinal cord injury in rats. Neurobiology of disease 32, 535-542.

Yu, P., Huang, L., Zou, J., Zhu, H., Wang, X., Yu, Z., Xu, X.M., Lu, P.H., 2007. DNA vaccine against NgR promotes functional recovery after spinal cord injury in adult rats. Brain research 1147, 66-76.

Zhan, R., Chen, S., Wang, W., Long, H., 2008. Effects of Nogo-neutralizing antibody and neurotrophin-3 on axonal regeneration following spinal cord injury in rats. Neural Regeneration Research 3, 1319-1323.

Zhang, Y., Gu, Z., Qiu, G., Song, Y., 2013. Combination of Chondroitinase ABC, Glial Cell Line-Derived Neurotrophic Factor and Nogo A Antibody Delayed-Release Microspheres Promotes the Functional Recovery of Spinal Cord Injury. The Journal of craniofacial surgery 24, 2153-2157.

Zhang, Y., Hao, C.G., Hu, L.Q., Dong, J., Wei, P., Xu, D., Xiao, Z.C., Wang, T.H., 2009. Recombinant DNA vaccine against inhibition of neurite outgrowth promotes functional recovery associated with endogeous NGF expression in spinal cord hemisected adult rats. Neurochemical research 34, 1635-1641.

Zhao, X.Y., Peng, Z.M., Long, L.L., Chen, N.N., Zheng, H.C., Deng, D.Y.B., Wan, Y., 2018. Lentiviral vector delivery of short hairpin RNA to NgR1 promotes nerve regeneration and locomotor recovery in injured rat spinal cord. Scientific Reports 8.

Zheng, B.H., Ho, C., Li, S.X., Keirstead, H., Steward, O., Tessier-Lavigne, M., 2003. Lack of enhanced spinal regeneration in Nogo-deficient mice. Neuron 38, 213-224.

Zhilai, Z., Hui, Z., Yinhai, C., Zhong, C., Shaoxiong, M., Bo, Y., Anmin, J., 2011. Combination of NEP 1-40 infusion and bone marrow-derived neurospheres transplantation inhibit glial scar formation and promote functional recovery after rat spinal cord injury. Neurology India 59, 579-585.

Supplemental Figure 1: Egger regression graph for detection of publication bias

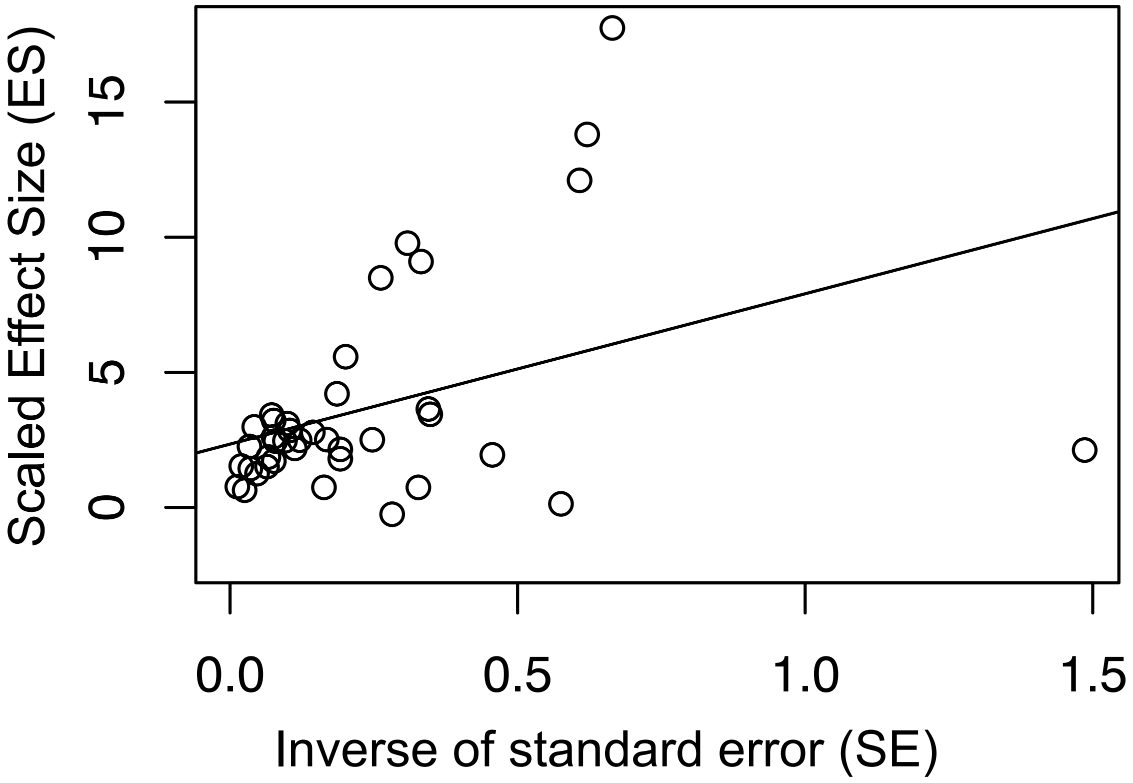

Supplemental Figure 2: Graphical abstract reflecting the evidence synthesis approach

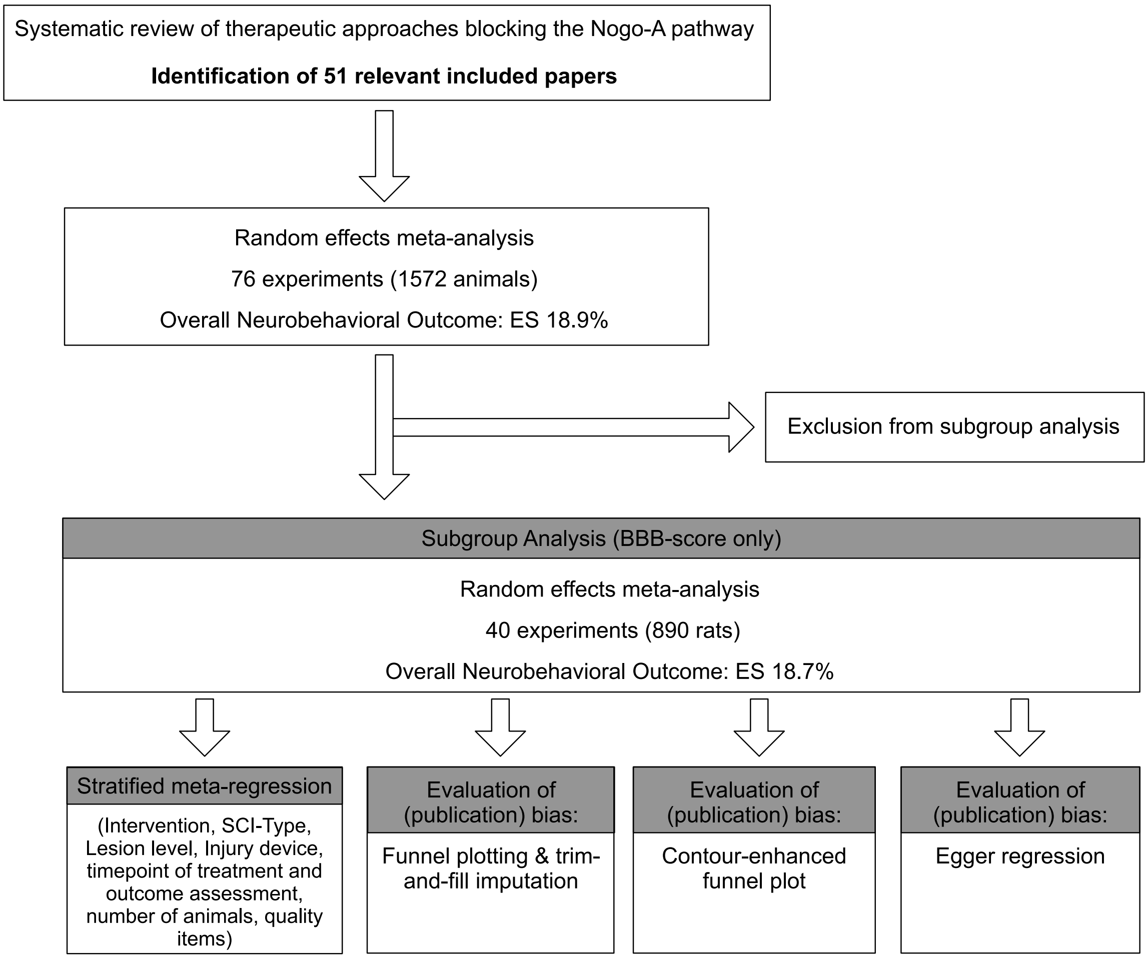

Supplement: Supplementary file 1 — Supplementary Information. [file 41598_2023_49260_MOESM1_ESM.pdf]
